# Wastewater Valorization: Practice around the World at Pilot- and Full-Scale

**DOI:** 10.3390/ijerph18189466

**Published:** 2021-09-08

**Authors:** Anouk F. Duque, Riccardo Campo, Angeles Val del Rio, Catarina L. Amorim

**Affiliations:** 1 Associate Laboratory i4HB—Institute for Health and Bioeconomy, NOVA School of Science and Technology, NOVA University Lisbon, 1099-085 Lisboa, Portugal; af.duque@fct.unl.pt; 2 UCIBIO—Applied Molecular Biosciences Unit, Department of Chemistry, NOVA School of Science and Technology, NOVA University Lisbon, 1099-085 Lisboa, Portugal; 3 DICEA—Dipartimento di Ingegneria Civile e Ambientale, Università degli Studi di Firenze, Via di S. Marta 3, 50139 Florence, Italy; riccardo.campo@unifi.it; 4 Department of Chemical Engineering, CRETUS Institute, Universidade de Santiago de Compostela, Rúa Lope Gómez de Marzoa s/n, E-15705 Santiago de Compostela, Spain; mangeles.val@usc.es; 5 CBQF—Centro de Biotecnologia e Química Fina—Laboratório Associado, Escola Superior de Biotecnologia, Universidade Católica Portuguesa, Rua Diogo Botelho 1327, 4169-005 Porto, Portugal

**Keywords:** wastewater, valorization, recycling, reuse, resource recovery, sludge

## Abstract

Over the last few years, wastewater treatment plants (WWTPs) have been rebranded as water resource recovery facilities (WRRFs), which recognize the resource recovery potential that exists in wastewater streams. WRRFs contribute to a circular economy by not only producing clean water but by recovering valuable resources such as nutrients, energy, and other bio-based materials. To this aim, huge efforts in technological progress have been made to valorize sewage and sewage sludge, transforming them into valuable resources. This review summarizes some of the widely used and effective strategies applied at pilot- and full-scale settings in order to valorize the wastewater treatment process. An overview of the different technologies applied in the water and sludge line is presented, covering a broad range of resources, i.e., water, biomass, energy, nutrients, volatile fatty acids (VFA), polyhydroxyalkanoates (PHA), and exopolymeric substances (EPS). Moreover, guidelines and regulations around the world related to water reuse and resource valorization are reviewed.

## 1. Introduction

The water demand is increasing worldwide. In fact, the world population is expected to reach over 9 billion in 2050, which will increase the demand for natural resources. Consequently, the global water demand is estimated to increase by 55% in 2050, mainly due to the higher water needs for activities such as manufacturing, thermal electricity generation, and domestic use [1]. As such, wastewater treatment plants (WWTP) have an important role in the safe return of water into the water cycle. In Europe, the adoption of the Urban Waste-Water Treatment Directive in 1991 (Council Directive 91/271/EEC) promoted the improvement of urban wastewater treatment, increasing the building of collection facilities and the proportion of the population connected to them. In fact, in 2017, for most of the EU-27 countries, at least 80% of their national population was connected to urban WWTPs. The main purpose of WWTPs is to produce high-quality water to be discharged into the environment. For that purpose, the chemical composition, health-related pollutants (e.g., pathogens), and aesthetic qualities of the treated water must be considered to minimize the environmental impact of the treated wastewater discharge. Although the traditional importance of WWTPs was to protect the environment and population health and wellbeing, in recent decades, the need for more sustainable processes has led to a paradigm shift in the water sector consisting of transforming wastewater from an unwanted waste stream to a valuable resource [2]. The current needs of modern societies have encouraged the scientific community to search for a wide range of technological solutions that have allowed for more circular resource flows, which aim at the resource recovery (e.g., water, nutrients, energy, and biomaterials) from wastewater. This transition to a circular economy model promotes a reduce-reuse-recycle strategy for waste management, contributing for the 2030 Agenda for Sustainable Development [3]. Therefore, the concept and term “WWTP” is being replaced by “WRRF” (Wastewater Resource Recovery Facility) nowadays [4].

The adoption of the circular economy principles in wastewater management can promote resource recovery and reuse, which can transform sanitation from a costly service to one that is more self-sustainable while adding value to the economy. To raise awareness on this issue among the stakeholders and practitioners involved in wastewater planning, financing, and management (e.g., water utilities, policy makers, or even ministries of planning and finance), a report was launched by the World Bank Water Global Practice Initiative that highlighted the different actions that need to be adopted [5]:

(1) Develop wastewater initiatives as part of a basin planning framework to maximize benefits, improve efficiency and resource allocation, and engage stakeholders;
(2) Build utilities of the future by shifting away from WWTPs to WRRFs, thus realizing the value of wastewater;
(3) Explore and support the development of innovative financing and sustainable business models in the water sector;
(4) Implement the necessary policy, institutional, and regulatory frameworks to promote the paradigm shift.

**Figure 1:**
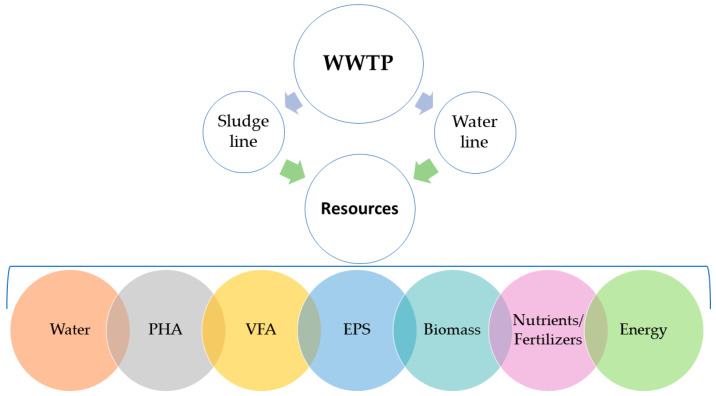
General overview of the review article: from wastewater to resource.

Resource recovery from wastewater treatment technologies at a larger scale is still scarcely reported. In this context, this review paper aims to provide a global and comprehensive view of the status of resource recovery at pilot- and full-scale WRRFs worldwide, with special focus on water recovery as the main valuable compound and also highlighting the importance of recovering other resources, namely biomass (as microalgae and single cell protein), energy (as biogas), nutrients (as fertilizers), or more novel biomaterials such as volatile fatty acids (VFA), polyhydroxyalkanoates (PHA), and exopolymeric substances (EPS) (Figure 1). Apart from the resources recovered from wastewater that will be described in this review paper, there are reports on the literature on the recovery of other types of resources with industrial interest, namely metals, sludge-based adsorbents, materials for the building sector, and proteins and enzymes [6,7,8,9] that will not be included in this review paper.

**Figure 2:**
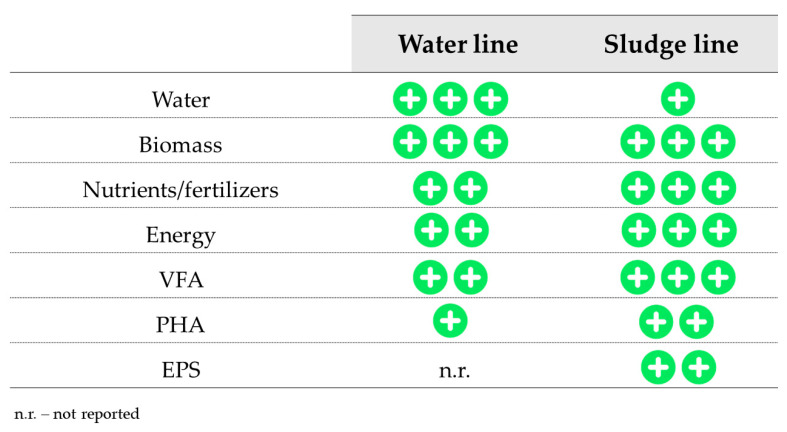
Summary of the resources recovered from wastewater reviewed in this study, with indication of the relative abundance of routes applied to the water and sludge lines.

The treatment of wastewater by traditional (activated sludge) or novel technologies (such as biofilm systems such as granular sludge) based on the use of biological processes is often preferred/adopted because of their cheaper operational expenditures (i.e., no chemicals required) and their lower environmental impact in comparison to physico-chemical processes. For this reason, the recovery of valuable products in biological-based processes can be made directly from the sewage (water line) or indirectly from the waste sludge that is produced (sludge line). Based on the information collected in the literature for the target recovered products, a comparative analysis between the routes applied to the water and sludge lines is provided (Figure 2). The feasibility of the technologies applied at a larger scale to recover added-value products differs between the water and sludge lines (Figure 2). Usually, it seems to be preferable to concentrate in the sludge the organic (i.e., VFA, PHA, EPS) and the inorganic (i.e., nutrients) compounds present in the wastewater to then obtain the biomaterials and nutrient-enriched streams from that sludge.

## 2. Water

The difference between water availability and water demand has caused water stress in different regions around the world, especially in those areas where water is scarce. For this reason, the recovery of water after its use is of extreme importance (water reclamation). Treated wastewater is one of the most available water resources that is constantly being produced and, its use for certain activities can considerably reduce the consumption of potable water. Reclaimed water can be used for various purposes including agriculture (irrigation), industrial processes, or even as drinking water after the application of a proper treatment chain (tertiary treatments, advanced oxidation process, ultrafiltration, reverse osmosis, etc.) in order to avoid the presence of pathogens. However, the most common use is for irrigation because of the stringent limits imposed for the other uses and the high volumes of water needed for this activity. In fact, according to the Food and Agriculture Organization of the United Nations (FAO), agriculture accounts for approximately 70% of all of the water withdrawn from natural water resources, which is much higher than the amount observed for other sectors (20% industrial and 10% municipal) [10]. Therefore, even if all of the industrially and municipally consumed water was reused, it would only cover approximately 43% of the amount needed for agriculture. For this reason, the reuse of wastewater for drinking water or industrial applications is not as cost effective as it is for irrigation. However, in the review of Ofori and colleagues [11], the inconveniences of wastewater reuse for irrigation are analyzed and highlight, for example, the impacts on the soil (pH and salinity changes, heavy metals accumulation, etc.), public health problems related to the presence of potential pathogens, and the presence of emerging contaminants such as microplastics, antibiotic resistant genes, and antibiotic resistant bacteria.

The greatest obstacles for wastewater reuse are related to technical (because it is more complicated to treat wastewater than it is to catch water from natural resources), economical (because wastewater treatment is normally expensive), social (because it is perceived as being more risky than beneficial), and political (because, for example, of the trade barriers for food products grown with reused water) issues [12]. Despite these obstacles, some successful examples of municipal and industrial wastewater reuse can be found around the world, which can serve to encourage new initiatives, especially in those regions where water scarcity will be a problem in the future.

### 2.1. Municipal Wastewater Reuse

One of the most encouraging processes for wastewater reuse was the pioneering program launched in 1968 in the city of Windhoek, in Namibia, which is the first city in the world to recycle wastewater for drinking water purposes [13]. In this city, the lack of water resources nearby, the low rate of rainfall (approximately 360 mm/year), the high rate of evaporation (ca. 3700 mm/year), and the continuous population growth (around 5% year) have forced the acceptance of alternative water sources, such as reclaimed water. Throughout the years, in order to meet the water demands due to the growing population, the city has been continuously adopting alternative strategies to increase the existing supply sources [13].

Israel is one of the countries with the highest water scarcity in the world. Currently, to alleviate this situation, desalination systems are used but, the use of reclaimed water has also emerged as an alternative source, satisfying around 25% of the country’s necessities [14] In fact, Israel recycles about 90% of used water, which is about four times higher than that recycled in any other country. Most of this reclaimed water is used for irrigation, while 10% is used for environmental purposes (e.g., increasing river flow and fire suppression), and 5% is discharged into the sea. Shafdan, in Tel Aviv, is the largest WWTP in Israel (average daily flow of about 360,000 m^3^/d in 2014) [14]. The treatment consists of an extended aeration activated sludge system with biological nitrogen removal, producing a high-quality effluent with average values of <6 mg BOD/L, <6 mg TSS/L, <6 mg TKN/L, and <1 mg TP/L. This effluent is infiltrated into a sand aquifer with an average retention time of 1 year, and it is then pumped and reused for unrestricted agricultural land application, covering more than 70% of the irrigation needs in that zone [15].

Other examples to illustrate wastewater reuse opportunities are presented in Table 1, which summarizes the main findings of several cities worldwide [16]. In all of these cases, the most significant motivation for wastewater reuse is water scarcity, but there are also concerns about preventing the pollution of water sources (by reducing the discharge of untreated wastewater) as well as about the recovery of added-value products such as energy and fertilizers. Although the proportion of wastewater reuse is relatively low in some cases (between 5–50%), there are cities such as Aqaba (69%) and Kampala (100%), which can serve as guides, opening prospects for other cities [16].

### 2.2. Industrial Wastewater Reuse

The use of reclaimed water in industrial applications is often limited to its use in the same industry. The particularities of some industrial processes lead to the presence of some compounds in the produced effluents, preventing their use in some applications. The major benefits of wastewater reuse for the industries are economical, as reuse contributes to the reduction of the costs of potable water consumption and additional effluent treatment processes needed to fulfil the legal discharge limits, and social, because it increases water availability in the surrounding community and increases the environmental awareness, improving the social perception of the industry [17].

Some industries can directly reuse wastewater whenever it is clean enough for the purpose for which it is being reused. For instance, the water used in cooling and heating processes normally contains low levels of contaminants. Thus, its reuse in the same industry for irrigation, washing, pH adjustment, or fire protection activities is feasible after a proper disinfection treatment in order to remove hazardous microorganisms such as legionella [18]. Nevertheless, a high percentage of water used in industrial processes cannot be reused directly and needs to be subject to a previous treatment. There are successful cases of wastewater treatment for its subsequent reuse in different industrial sectors (Table 2).

The land-based aquaculture sector is perhaps the most advanced sector in terms of reusing water with the application of recirculating aquaculture systems (RAS), which have been steadily developed over the past 30 years to minimize the water withdrawn, to control culture conditions, and to allow waste streams to be fully managed [23]. Yet, the recirculating aquaculture systems still need to address some challenges, such as the complex and costly engineered system designs [24].

Recently, the integrated Industrial–Urban Water-Reuse (IU-WA-RE) concept has attracted great attention [25]. This approach aims to substantially increase water-reuse by combining industrial and municipal wastewater flows. The high loads of wastewater generated in industrial parks after being adequately treated can then be reused for the irrigation of the surrounding urban areas, as municipal wastewater is usually not enough to cover the water requirements for urban areas for infrastructural purposes [25].

### 2.3. Water Reuse Legislation

In Europe, only a small proportion of treated wastewater is reused (1100 million m^3^/year, while the potential is around 6000 million m^3^/year), and the main application for its reuse is for irrigation purposes [26]. In fact, treated wastewater can be an important water source for agriculture, but its application should be carefully regulated to hinder the use of water with insufficient quality that can later pose danger to human health. From 1980 to 2020, the European Union has thus developed different directives of major importance for the water sector to protect the environment and human health and to regulate the water cycle. The first directives launched by the European Union aimed at protecting the environment from the adverse effects of urban wastewater discharge. In 2000, the Council Water Directive (2000/60/EC) highlighted the need for more sustainable water usage for the first time. It was only in 2020 that the European Union launched the regulation on water reuse, stating the minimum requirements for water reuse (Regulation (EU) 2020/741) [27]. However, some authors have criticized that this regulation does not sufficiently cover relevant risks to protect human and environmental health, and several key aspects were inadequately addressed, for example, concerns surrounding contaminants of emerging concern, the spread of antibiotic resistance, and disinfection by-products [28].

In the United States, there is a general framework under the Safe Drinking Water Act [29] and the Clean Water Act [30] to regulate water reuse. In fact, the Environmental Protection Agency (EPA) does not require or restrict any type of reuse, but the water stream that is to be reused should be adequately treated to meet “fit-for-purpose specifications” for its specific next use [31]. More recently, a National Water Reuse Action Plan (WRAP) was developed, which included specific actions to improve the use of reclaimed water in the nation, which only accounts for 1% of the water demand [32]. The latter indicates that reuse can cover different applications apart from irrigation, such as potable and non-potable water supplies, groundwater storage and recharge, industrial processes, onsite non-potable use, saltwater intrusion barriers, and environmental restoration. The newly proposed actions were classified into eleven strategic themes [33]. Among others, “Science and Specifications” and “Technology Development” can be highlighted, where the salinity of reused water is one of the main concerns because salts are normally not removed in conventional WWTPs, and their presence further limits applications such as irrigation (Table 3).

One of the states where the water scarcity forces water reuse is California. In this state, the volume of recycled water used has more than doubled in the last two decades, but water demand still exceeds supply [34]. If the energy use per water source is considered, in California, the water produced by wastewater treatment is cheaper than that produced from seawater or groundwater desalination systems [35].

Even though there are some constraints, the safe reuse of the treated wastewater, if well-managed, can significantly reduce potable water demand, thus contributing to more sustainable water use.

Australia is one of the regions in the world where water scarcity strongly influences water reuse, as pointed out in the review by Radcliffe and Page [36]. These authors revised the past, present, and future perspectives for water reuse in that country, highlighting the strong influence of drought periods and policies that have been adopted. For example, the millennium drought (2000–2009) boosted water recycling and desalination, with the construction of advanced purified recycled water plants for indirect potable reuse as well as dual pipe installations for drinking and recycled water. However, after the drought, the catchment of surface water was cheaper than recycled or desalinated water, and economic motivations decreased its reuse. The return of drought conditions in 2019 saw the desalination systems reactivated, and although indirect potable recycling schemes were adopted again, community debate on direct potable recycling needs to be addressed.

## 3. Biomass

Huge amounts of sludge are annually produced in WWTPs, which can represent an environmental problem if the sludge is not correctly disposed of. Moreover, its disposal also represents additional costs for WWTPs, as it is estimated that the costs of its processing accounts for approximately 50% of overall WWTP operating expenses [37]. Waste sludge is usually a heterogeneous solid material consisting of water, microorganisms, organic matter, inorganic, and organic compounds [37]. This composition thus offers great potential for valorization, which can then turn waste sludge disposal from a major cost into a source of profit for WWTPs. According to European Parliament and Council Directive 2008/98/EC [38], the reduction of waste production should be the priority in sludge waste management, followed by the use of waste for reuse, recycling, or other forms of recovery, and ultimately, only sludge waste disposal should be considered. To assist the wastewater sector to meet this waste management hierarchy, several strategies have emerged that allow the production of a set of different marketable outputs from waste sludge. Effective strategies to achieve sludge valorization include its use as a product or as a source for resource recovery, such as for nitrogen, phosphorous, or carbon (e.g., in the forms of VFAs, PHAs, or methane (CH_4_)), and examples of these processes will be addressed in the following sections. The valorization of waste sludge through the production of added-value products is an important step towards a more sustainable society, with the expectation being that in the near future, WWTPs will be able to be converted into biorefineries in which the “production” of treated water is no longer the sole requirement.

### 3.1. Microalgae Biomass

Microalgae based systems are ecofriendly and sustainable wastewater treatment options due to their effective capacity to treat both municipal and industrial wastewater while also allowing CO₂ fixation, saving nutrient input, and producing microalgal biomass, which is a source of a myriad of value-added algae-derived bioproducts and biomaterials [39].

Several research works at laboratory-scale have demonstrated the extraordinary capabilities of microalgae to treat wastewater of various origins, either directly or after pre-treatment processes [40]. More recently, microalgal-bacterial sludge based processes have attracted increasing attention due to the mutualistic and symbiotic relationship that microalgae and bacteria can establish [41]. The oxygen produced via photosynthesis by the microalgae cells can be used by the bacteria to oxidize organic matter, thus allowing for the collaboration between the microalgae and aerobic bacteria in the same system. This is particularly promising, as it can decrease the costs of aeration and can also reduce carbon dioxide emissions.

Although there have been encouraging results for wastewater valorization using microalgae-based systems at laboratory-scale, outdoor pilot- and full-scale studies are still scarce, and obviously, there is a long journey ahead for the large-scale application of these systems. Nevertheless, research efforts have been made to assess the scale-up potential of microalgae cultivation within a conventional wastewater treatment sequence in outdoor photobioreactors (Table 4).

The possibility of including microalgae culturing using microalgae-based systems for the treatment of the side stream flow of centrate from biosolid dewatering has been largely explored. Centrate has a high nitrogen concentration; thus, it cannot be directly discharged. Thus, this stream is usually sent back to the water line of the WWTP to be further treated, increasing the nitrogen load at the entrance of the biological processes in 20–30% [42]. The main aim of this process is to produce microalgae biomass that can be further used to feed the existent anaerobic digesters to increase biogas production and, in turn, improve the energy balance and the carbon footprint of the whole process. Additionally, the oxygen produced by microalgae will potentially reduce the aeration demand for nitrification in the water line. The microalgae biomass productivities found in the different pilot-scale studies using centrate streams were very variable, which is probably related to the environmental conditions and to the centrate composition or may even be due to the type of reactor used [42,43,44,45,46,47]. Nevertheless, in the long run, it seems to be a feasible integrated biorefinery process.

Owing to the microalgae biomass applications in diverse fields, there is a huge prospect for the development of sustainable processes where microalgal production can be accomplished using industrial wastewater. Kumar and colleagues [50] evaluated the techno-economic feasibility of a microalgae-based dairy effluent treatment for the simultaneous production of microalgae biomass and clean water. The high volume V-shaped pond used in this study seemed to be a cost effective and area efficient microalgal cultivation system, allowing an annual algal production of 504 ton at USD 0.482/kg along with a production of approximately 240,000 m^3^ of treated clean water. Recently, in a circular economy context, the potential of polishing of swine wastewater with microalgae along with the production of biomass with added value was explored [52]. At the beginning, the bacterial activity exceeded that of the photosynthesizing organisms, but in the long run, the proportion of photosynthesizing organisms in the total biomass substantially increased.

### 3.2. Single Cell Protein

The increasing demand for food supply, especially proteins, forced the search for new ways to obtain them. Recently, there is an increasing interest to obtain single cell proteins (SCP) by recycling the organics and nutrients present in wastewater. Sewage sludge is a valuable source of proteins due to the high content of these components in the sludge composition (about 61% proteins, 11% carbohydrates, 1% lipids, and 27% other components) [54]. Taking in account that approximately 50% of the dry weight of the bacterial cells are proteins, the protein extract that can be recovered from sewage sludge is very promising. Nevertheless, apart from the bacterial cultures, the SCP can also be obtained from other types of biomasses such as microscopic algae, yeast or fungi [55]. Some examples of SCP production from different streams are shown in Table 5.

Phototrophic organisms, such as microalgae or purple phototrophic bacteria, appear as the best solution because of their high carbon yields and high nutrient capture potentials [59]. In the research work of Hülsen and colleagues [60], the capacities of microalgae and purple phototrophic bacteria to treat different agro-industrial wastewaters and to produce SCP were compared. They concluded that the removal efficiencies (COD, Nitrogen and Phosphorous) were better with microalgae than with purple phototrophic bacteria, but the latter had better SCP production (>50% protein content) than microalgae (<30% protein content). In fact, in the review of Capson-Tojo and colleagues [61], it is stated that the biomass yield of purple phototrophic bacteria can range between 0.5 and 1.0 g COD/g COD _removed_, which indicates the high potential of this type of culture to obtain SCP. Technologies based on purple phototrophic bacteria are starting to be implemented at the pilot-scale, and there is still no available data for larger-setting applications.

Regarding legislation for the use of SCP obtained from waste, such as wastewater, the review from Hülsen and colleagues [55] states that there are several guidelines and regulations around the world with complex and case-dependent situations to commercialize this type of product. The different legislations differ between food for humans and for animals, and in this last category, between pets and livestock. There are also differences between food, feed additives, and medical feed. Therefore, the presence of pathogens, xenobiotics, metals, etc., which are present in most of the wastewater streams from municipal WWTPs, causes the production of SCP to be preferentially applied to wastewater streams generated by the food processing industries [55].

## 4. Nutrients and Fertilizers

Nutrient (Nitrogen and Phosphorous) recovery as soil fertilizers from wastewater can be a good alternative to avoid unregulated wastewater discharge while also solving the problem of poor access to chemical synthetized fertilizers, especially in developing countries, due to their high cost. 

Phosphorous is a nonrenewable natural resource that is essential for the production of phosphorous-based fertilizers. The lack of this resource in the planet led to the development of biological and chemical processes to recover nutrients from wastewater and sludge [62,63]. In the review of Saliu and Oladoja [64], the authors stated the possibility of nutrient recovery from different kinds of nutrient-rich wastewater including that from agricultural practices and from industrial and municipal facilities. Additionally, the reviewed literature confirmed the viability of the recovered nutrients for reuse as fertilizer in agricultural practices, as their fertilizing effect is comparable with or is even better than commercial fertilizers. Some examples of nutrient recovery from wastewater as valuable products at full-scale are presented in Table 6.

Considering the high cost of ammonium removal in conventional WWTPs by nitrification-denitrification processes and of ammonium-based fertilizer production by the Haber–Bosch process, the recovery of such compound seems more valuable than its removal. However, ammonium recovery is only economically feasible when applied to large WWTPs that are able to produce large concentrations of such ions [68]. The ammonium concentration in wastewater sources varies depending on the origin. For example, in municipal WWTPs, the mainstream line contains around 100 mg of nitrogen per litre, while the reject water from anaerobic sludge digesters has around 1000 mg of nitrogen per litre. According to Ye and colleagues [68], there are three main ammonium recovery mechanisms: struvite precipitation, ammonia stripping coupled with adsorption, and membrane concentration. The struvite precipitation is more advantageous as, apart from ammonium, it simultaneously recovers phosphate. The struvite (magnesium ammonium phosphate hexahydrate) is often preferable over mineral phosphorous fertilizers, as it is less soluble in water, and when applied to soils, it allows the slow release of phosphorous, which is more beneficial for plant growth [69]. Recovery by means of the stripping-adsorption mechanisms need the application of high temperatures and/or pH values, and the stripped ammonia is adsorbed by acid solutions to form ammonium salts such as ammonium sulphate. Alternatively, through the membrane concentration, ammonium enrichment can be accomplished, separating it from the other co-existent substances. In this case, the membrane technology involved can be forward osmosis, reverse osmosis, membrane distillation, and electrodialysis.

Regarding the phosphorous present in wastewater, about 95% is transferred to the sludge in WWTPs. Phosphorous can be recovered in multiple sections of a WWTP through three main paths: from the liquid phase (i.e., in aqueous phase from digester supernatant, as dissolved phosphorous in anaerobically digested sludge, and in the effluent), from sewage sludge (direct agricultural utilization), and from sewage sludge incineration ashes [70]. Therefore, to date, various technologies have been applied for phosphorous recovery at pilot- and full-scale levels in WWTPs, namely the REM-NUT^®^ (ion exchange and precipitation in the secondary treated effluent), AirPrex^®^ (precipitation/crystallization of dissolved phosphorous contained in the anaerobically digested sludge), Ostara-Pearl-Reactor^®^, DHV Crystalactor^®^, P-RoC^®^, PRISA^®^ (all these technologies are based on crystallization of phosphorous in digester supernatant), Gifhorn and Stuttgart processes^®^ (wet chemical leaching from digested sludge), PHOXNAN^®^ (wet oxidation of thickened sludge), Aqua Reci^®^ (super critical water oxidation of thickened sludge), MEPHREC^®^ (metallurgic melt-gassing of dewatered sludge), AshDec^®^ depollution (thermo-chemical depollution of sludge ash), AshDec^®^ Rhenania (thermo-chemical phosphorous recovery from sludge ash), PASCH^®^, LEACHPHOS^®^, EcoPhos^®^ (acidic wet-chemical leaching of sludge ash), RecoPhos^®^, Fertilizer Industry^®^ (acidic wet-chemical extraction of sludge ash), and Thermophos (P_4_)^®^ (thermo-electrical phosphorous extraction) [70]. However, a critical issue with phosphorous recovery processes is mainly related to the presence of metals (e.g., Zn, Cu, Cd) in the final products. The removal of metals from the final product is strictly dependent on the local regulatory requirements, and it is mandatory in the case of fertilizer production. This obviously affects the phosphorous recovery costs [70].

In Europe, wastewater nutrient recovery as fertilizers had not a dedicated legislation until 2019, when the Fertilizing Products Regulation 2019/1009 was published. This regulation states that the impurities in European Union fertilizing products derived from biowaste, particularly from metals such as cadmium, should be either prevented or limited.

In the United States, the use of sewage or sewage sludge as fertilizer in agriculture is regulated under the Clean Water Act and, more specifically, in the 40 Code of Federal Regulations Part 503 “Standards for the Use or Disposal of Sewage Sludge” [71]. These regulations stablish general requirements, pollutant limits (specially for metals), management practices, and operational standards for the final use or disposal of sewage sludge.

## 5. Energy

The nexus between water and energy is one of the crucial elements for sustainable development. As WWTPs consume large amounts of energy, research efforts are underway to develop technologies for its recovery and to convert WWTPs from energy consumers to energy producers. The types of energy that can be obtained from sewage include biogas from anaerobic digestion (the most common), electrical energy (from bioelectrochemical treatment processes), low-head hydroelectric energy, renewable fuels from sludge processing, and heat energy [72].

Anaerobic digestion consists of the degradation of complex organic matter that is converted into biogas, such as methane and carbon dioxide, which can then be energetically revalorized. Traditionally, anaerobic digestion is performed by a synergic microbial consortium in four sequential steps (hydrolysis, acidogenesis, acetogenesis, and methanogenesis), where the products resulting from one stage are then used as substrate for the following stages and ending up in biogas production [73]. 

Conventional WWTPs commonly include activated sludge treatment to treat the incoming wastewater plus the anaerobic digestion process for the surplus sludge that is produced, which are well established processes in WWTPs from many countries [74]. Anaerobic digestion is therefore one of the most commonly applied bioprocesses for recovering energy from sewage sludge (Table 7).

Nevertheless, it is also applied to recover energy directly from industrial and municipal wastewater; the latter is mainly the case in countries with warm temperatures, such as Brazil [75].

A major drawback in recovering energy from sewage sludge is related to the low biodegradability of the sludge produced in urban WWTPs, which often leads to low biogas (methane) yields. Therefore, the anaerobic co-digestion of wastewater surplus sludge and other biomass wastes has thus emerged as a viable alternative to increase biogas production. The co-digestion of sludge and food waste has been explored, as it contributes to the circular economy concept: products at the end of their life service or waste materials are turned into resources for another valuable purpose, thus closing loops in industrial ecosystems and minimizing waste [76].

Recently, Li and colleagues [83] explored an integrated process to valorize sewage sludge, aiming at its full use, in a process combining hydrothermal pretreatment, anaerobic digestion, and pyrolysis. This pilot-scale study showed that the hydrothermal pretreatment of sewage sludge improved the dewaterability of the sludge, generating a filter cake with a solid content of 67% wt while the continuous anaerobic digestion of the resulting filtrate presented a methane yield of 260 mL/g COD, a quantity of biogas that is able to compensate for the energy required for the former process. Meanwhile, the filter cake was pyrolyzed to generate biochar.

Microalgae-based systems are being largely adopted in full-scale WWTPs, and the potential of microalgae biomass as feedstock for biofuels is very appealing due to their higher heating values and rapid growth rate. Microalgae gasification using compact microgeneration systems was successfully explored at pilot-scale as an alternative for the microgeneration of energy in WWTPs [84]. A commercial downdraft gasifier was used, and the best performance obtained a syngas production rate of 2.8 Nm^3^/kg biomass dry, with a syngas composition of 11.9% H_2_, 19.5% CO, 8.5% C_x_H_y_ and 9.8% CO_2_.

Another promising alternative for energy generation are microbial fuel cells (MFC). In this case, the organic load present in the wastewater is converted into electrical energy by bacteria. The MFC are a very promising technology, but its scale up has faced some challenges due to the complexity of the installation and operating procedures as well as other engineering and environmental factors [85]. One of the main problems is that power generation decreases with the increase of the reactor size. Miniaturization of the MFC and the connection of multiple MFC units in stack configurations were adopted strategies that allowed for increased power densities. Perhaps these challenges hindered the investigation of large-scale prototypes, as it has only been more recently that pilot studies have been conducted (Table 8). Most of these pilot studies explored the use of stackable or tubular units to multiply MFC components. By connecting those multiple small-sized units, power generation can be largely increased, thus contributing to the technology applicability to meet the criteria for high performance in real-world conditions.

The possibility of using wastewater as an alternative source of energy for heating has recently emerged as a feasible approach. Cecconet and colleagues [72] designed a system with heat exchangers and pumps where the energy contained in the wastewater was recovered for heating and cooling a building with a calculated energy requirement of 957 MWh per year, which allowed for a reduction of 59% of this value.

## 6. Volatile Fatty Acids

**Figure 3:**
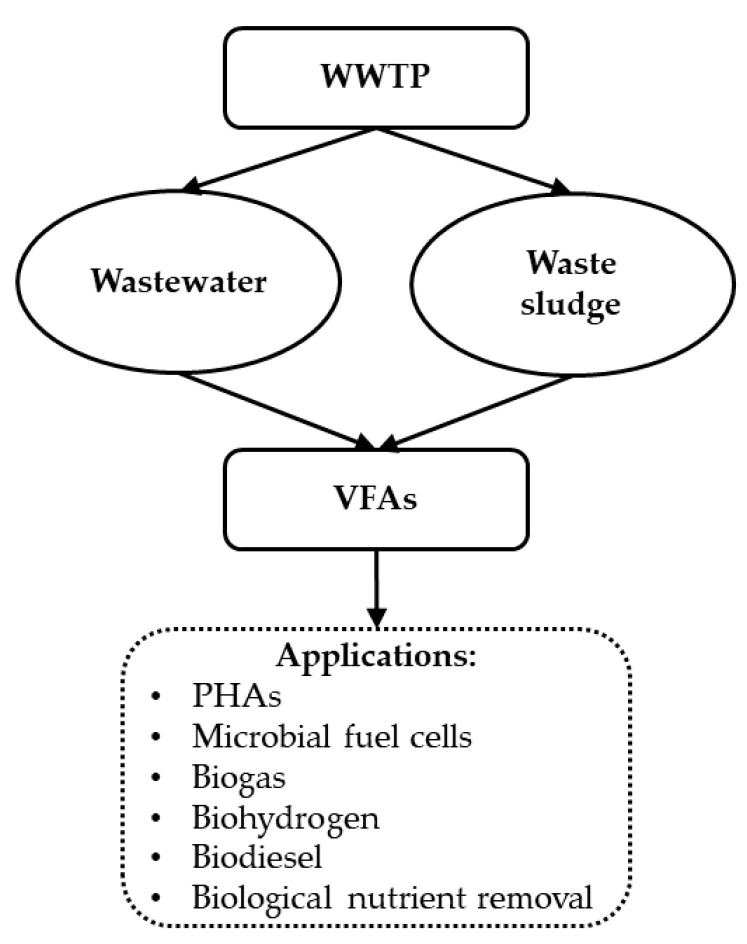
Biorefinery concept overview: volatile fatty acids production and their applications.

The volatile fatty acids (VFAs) are valuable compounds with a high market demand and with several applications, namely as precursors for bioplastics (e.g., polyhydroxyalkanoates (PHA)), biogas, biohydrogen, and biodiesel production as well as for nutrient removal (Figure 3) [91,92]. 

VFAs also have high industrial interest as chemical building blocks, such as for use as plasticizers, food additives, dyes, resins, pharmaceuticals, and paints. VFAs are short-chain fatty acids with a low molecular weight that consist of two to six carbon atoms and that are mostly derived from fossil fuels using chemical routes, leading to serious negative health and environmental impacts [93,94]. Thus, the replacement of these processes by biological ones such as by using pure or mixed microbial cultures or by using renewable carbon sources is gaining more attention [91,92,94,95]. This strategy represents a cost-effective and environmentally friendly alternative for VFAs production [91]. The use of pure cultures for VFA production has been studied extensively [91]. However, such processes use refined carbon sources and must occur under sterile conditions, leading to high operating costs. Thus, research on mixed microbial cultures is emerging [91,94,95,96]. Mixed microbial cultures can use organic waste as carbon source, such as sewage or sewage sludge, with the added advantage that they do not need sterile conditions to operate. This presents several advantages, namely decreasing generated waste and contributing to environmental sustainability and to a circular economy.

The conversion of the organic content of waste feedstock into VFAs by mixed microbial cultures involves an acidogenic fermentation process [97]. This is an anaerobic process that involves both hydrolysis and acidogenesis. In hydrolysis, complex organic polymers (such as proteins, cellulose, lignin, and lipids) are broken down into simpler soluble monomers (such as amino acids, simple sugars, glycerol, and fatty acids) by the enzymes excreted by hydrolytic microorganisms. Then, these monomers are converted into VFAs (acidogenesis) by fermentative acidogenic bacteria. In an anaerobic reactor, both processes occur simultaneously, with hydrolysis generally being considered the rate-limiting step. To avoid the consumption of VFAs for biogas production by methanogens, a high pH (above 8) or low pH (below 6) is generally used [95,98,99,100]. Moreover, to maximize VFA production yield and to control the composition of the synthesized VFAs, operating parameters such as hydraulic retention time (HRT), sludge retention time (SRT), organic loading rate (OLR), pH, temperature, and reactor configuration must be optimized.

Although most of the studies on VFA production using biological routes are at lab-scale, some pilot- and full-scale studies with promising results can be found in the literature and are summarized in Table 9. 

Even though specific regulations on the application and commercialization of VFAs derived from waste and wastewater is lacking, Directive 2008/98/EC of the European Parliament and of the Council of 19 November 2008 states measures for the handling and management of waste [38]. This Directive encourages the use of environmentally safe materials produced from biowaste, which may include VFAs produced from waste and wastewater. In the near future, legislation regulating the products from the valorization of waste and wastewater will certainly appear as a response to the need for sustainable alternatives.

## 7. Polyhydroxyalkanoates

Polyhydroxyalkanoates (PHAs), a family of polyesters that are naturally produced by bacteria as energy and carbon storage materials, are an alternative to conventional fossil-based plastics, and their production and accumulation in the planet are harmful to the environment and human health [104]. These bioplastics are biodegradable and biocompatible, can be produced from renewable resources, and have similar properties to petrochemical polymers, namely propylene [97,105,106]. Due to their biocompatibility, biodegradability, and green credentials, PHAs are being extensively applied in many fields, namely in the medical sector (e.g., tissue engineering, bio-implant patches, drug delivery, surgical applications, medical devices) and in nanotechnology (e.g., biocomposites for applications in various industrial sectors, such as in packaging, agriculture, automotive industry, and building) [107,108,109].

Industrial processes for PHA production are currently based on pure cultures and pure substrates (e.g., Biomer-Germany, Tianan-China). Making this process more cost effective and competitive, low cost processes for PHA production, such as using mixed microbial cultures, have been developed [110,111]. The use of mixed microbial cultures instead of pure cultures allows for the reduction of PHA production costs, and since no sterilization is necessary, few process controls are required, and cheap or even free substrates such as industrial waste or by-products can be used as feedstock since the microbial population can continuously adapt to changes in the substrate [97,112,113,114,115,116,117]. Municipal wastewater sludge can be a sustainable alternative as it is a raw material that can be used to produce bioplastics, namely as a substrate or as a source of PHA accumulating microorganisms [118].

PHA production processes involving mixed microbial cultures commonly operate in 3-stages [97]: (1) the acidogenic fermentation stage, (2) the culture selection stage, and (3) the PHA production stage. The acidogenic fermentation process enables the conversion of the organic content of waste feedstock into VFAs, which are the preferred substrates for PHA production [97]. Control of the produced VFAs is extremely important since it will influence final polymer composition and, consequently, affect its thermomechanical properties [97,113,119]. 

In the culture selection stage (second stage), the mixed microbial cultures are subjected to alternate periods of substrate excess (feast) and limitation (famine) in a process known as Aerobic Dynamic Feeding or the feast and famine regime, which allows the enrichment in organisms with high and stable PHA storage capacity. It is considered that the transient external substrate availability is responsible for creating an internal growth limitation that will promote the PHA storage capacity of the mixed microbial cultures. Additionally, the second aim of this stage is the production of a culture with high biomass volumetric productivity (high growth rate) without the impairment of high storage capacity for the accumulation stage. However, unlike pure cultures, this is still a challenge [97,120].

In the PHA production stage (third stage), the selected mixed microbial cultures from the second stage are fed with the VFAs produced in the acidogenic fermentation stage, aiming to achieve the maximum PHA production capacity of the cultures. Even though PHA production from waste streams has been successfully applied at lab-scale, PHA recovery is still a costly step in the mixed microbial cultures process. Thus, it is still a challenge for the PHA production process up-scaling using mixed microbial cultures to become economically feasible [121,122]. However, some PHA producing pilot-scale plants based on the use of mixed microbial cultures and wastewater as a substrate can be found in the literature and are listed in Table 10. 

The Veolia Group, the global leader in optimized resource management, is now working on the development of full-scale industrial PHA production from domestic wastewater, and a pilot plant is already in operation in Belgium [123]. 

There are still some constraints in the commercial use of PHAs derived from waste due to a lack of legislation. Recently PHAs were not considered to be natural polymers and thus were not considered suitable for single-use plastic products (Directive (EU) 2019/904 of the European Parliament and of the Council of 5 June 2019 on the reduction of the impact of certain plastic products on the environment) [134]. This has led to some discussion, as PHA producers and researchers disagree with this definition and claim that this European Union Directive guideline can compromise PHA potential as a sustainable alternative to single use fossil-fuel derived plastics in the European Union (GO!PHA—Global Organization for PHA). However, it is expected that new legislation on sustainable options for traditional plastics, e.g., the application of products derived from waste and wastewater, will arise in the coming years as part of the Circular Economy Action Plan (European Commission 2020).

## 8. Extracellular Polymeric Substances

Extracellular polymeric substances (EPS) are believed to play an important role in the WWTP processes in the formation and stability of both sludge flocs and biofilms, as this self-produced hydrated matrix of large polymeric molecules envelopes bacterial cells [135]. Furthermore, EPS are currently considered as potential resources and thus are key players in the paradigm shift from WWTPs to WRRFs [136]. The EPS are located at the outside of the bacterial cells surface, and their production and composition are thought to be controlled by different processes, such as active secretion, the shedding of cell surface material, cell lysis, and adsorption from the environment [137]. The presence of EPS in sludge contributes to the aggregation of bacterial cells in flocs and biofilms (e.g., granular sludge), bacteria protection acting as barrier against harmful substances, the water-binding capacity, and the enzymatic action such as the digestion of complex macromolecules for nutrient acquisition [138]. In the past, biofilm research often assumed that polysaccharides were the predominant components of EPS [139]. However, proteins, humic acids, fulvic acids, and nucleic acids are also abundant in EPS from several sources [140,141,142].

The first studies on the recovery of EPS conducted on activated sludge biomass from full-scale WWTPs demonstrated some interesting properties of these biopolymers, such as their bioflocculation [143] and metal biosorption capacities [144,145,146]. More recently, these interesting properties have also been found in EPS extracted from granular biomasses such as aerobic granular sludge (AGS) and anammox granules.

Although conventionally activated sludge systems are still the most common technology used in WWTPs, systems based on granular sludge have been increasingly adopted worldwide, which is in part due to their high performance and lower footprint [147]. Past studies have revealed that a particular fraction of EPS, named structural EPS (sEPS), has characteristics similar to alginate polymers and, can be extracted from AGS with an yield in the range 20–30% wt as volatile solids (VS) [148,149]. A first comparison between the activated sludge or AGS recovered EPS can be drawn in terms of extraction yield, which is much lower in the case of activated sludge-based EPS (3.5–7.2% wt as VS) [150]. However, it should be noted that extraction of granular sludge-based sEPS requires more intensive and expensive extraction methods than that of flocs. Nevertheless, in both cases, sEPS extraction reduces the sludge volumes to be disposed (i.e., operational expenditure cost reduction), and the residual sludge could have higher digestibility [151]. The first large-scale EPS production unit is already in operation in Zutphen (The Netherlands), and a second production unit in Epe is projected [152]. The EPS, marketed as Kaumera Nereda^®^ Gum, is extracted from granular sludge originating from the Nereda^®^ wastewater treatment process. This process will ensure that 20–35% less sludge will need to be processed, thus reducing energy consumption and CO_2_ emissions. Based on the experimental value of 21.9% wt as VS of sEPS recovered from a pilot-scale AGS process treating municipal wastewater [153], it is expected that about 22.5% wt as VS of sEPS can be recovered from waste AGS [154]. More recently, the feasibility of recovering EPS from surplus AGS biomass was assessed in full-scale operational settings [155]. Although variations in the EPS concentration and composition were observed over time overall, it was estimated that the surplus biomass produced at that WWTP would allow for the recovery of 4–5 ton of EPS per day or 0.6–1 ton of purer EPS per day, depending on the composition variability that was aimed for.

Regarding the EPS extracted from anammox granular sludge, the extensive classification of glycoproteins originating from it allowed for the better understanding of their structure [156]. The protein and polysaccharide analyses from the extracted EPS revealed 599 mg/g VS _EPS_ and 49 mg/g VS _EPS_, respectively [138,157].

Bearing in mind the above, it should be stressed that EPS composition largely depends on various factors such as sampling, the type of wastewater sludge, the operating conditions, and the extraction methods, as shown in Table 11. More exactly, given the high diversity of the granular sludge-based EPS and the applied extraction and recovery methods (i.e., heating-alkaline conditions, acidic conditions, etc.), differences in terms of the yields, components, and functional groups of the EPS are observed [88,102,111]. However, the existing extraction and recovery methods have never been considered in terms of the recoverable material characteristics for specific practical applications and have only been aimed at maximizing the extraction yield. Hence, designing the proper extraction method looking at specific applications is an aspect that requires consideration when it comes to reusing the recovered EPS-based materials in specific industrial sectors.

Some recent studies have estimated that the full-scale recovered AGS-based EPS could reach 85 kton in the Netherlands in the coming decade [154,158]. Likewise, the anammox granular sludge-based EPS could reach 185 kg-EPS/d from full-scale partial nitritation/anammox processes [159]. 

EPS recovery will lead to a reduction in the amount of waste sludge to be disposed, thus promoting a paradigm-shift from WWTPs to WRRFs. Furthermore, the peculiar characteristics of the recovered EPS make them a resource that should be used as much as possible. Currently, there are numerous applications for the EPS recovered from waste biomass (Table 12). EPS could potentially be used in the chemical, agriculture, or building sectors, among others. 

The EPS recovered from granular sludge were found to be able to form hydrogels with peculiar rheological properties [136,166]. The hydrogel-forming capacity of sEPS allows for their potential application as an industrial paper coating to increase the waterproof properties [167,168]. The hydrophilic and hydrophobic functional groups present in EPS provide abundant binding sites, which are closely related to the hydrogel enhanced features. 

As AGS-based sEPS have hydrophilic characteristics, they can be commercially applied to improve the curing of cement [166]. The mechanism is related to reducing moisture loss from the surface of cement-based materials, which is fundamental in construction engineering [169]. The retention of cement surface humidity is very important to avoid structural cracking due to drying shrinkage. 

Additionally, granular sludge-based EPS have been proven to be a cost-effective biosorbent material for several water treatments, as they are able to bind metal ions (Ni^2+^, Pb^2+^, Cd^2+^) [170,171] or organic compounds [172]. Nevertheless, it should be stressed that the biosorption effectiveness of granular sludge-based EPS strictly depends on pH, temperature, conductivity, efficient contact area/time between EPS and pollutants, and pollutant structure and concentration [170].

Another property of AGS-based sEPS is regarding their possibility to be used as extinguished bio-based flame retardant materials for flax fabrics due to their effective char formation [173]. EPS have self-extinguishing properties, indicating their feasible application as coating materials [173]. To date, the market for flame retardants comprises almost 31% halogenated materials even though they have hazardous influences on humans and on the environment. In this context, the EPS extracted from granular sludge could be a “green” bio-based alternative, reducing the consumption of halogenated materials.

Recently, the potential application of EPS as bio-flocculants in place of synthetic polyelectrolytes was reported [174]. The EPS functional composition in this case plays an important role in the flocculation ability as the presence of some functional groups improve the agglomeration of particulate matter and floc formation.

A recent application of sEPS lies in the agronomic sector as a soil conditioner [175]. They can be applied as a bio-stimulant and as a slow-release fertilizer. sEPS can retain water (water-binding capacity) [138] and thus improve plant growth.

## 9. Conclusions

To meet the global challenges, e.g., increasing water demand, water shortage, and decreasing availability of non-renewable resources, the adoption of the so-called WRRF concept is crucial. Nowadays, sewage is no longer regarded as a waste but rather as a source of valuable resources, resulting in environmental and social-economic benefits. To date, the resource recovery concept has been successfully applied in pilot- and full-scale facilities. Wastewater treatment for reuse is indeed a plausible solution to combat the world’s water scarcity problem. The available technologies allow wastewater to be properly treated, producing effluents with quality that satisfies the demand from different sectors, including industry and agriculture. However, scientific advances have shown that wastewater valorization is not limited to water reuse, recognizing that wastewater contains several valuable resources that can be recovered. Concerning the excess of sludge produced during wastewater treatment, several strategies can be applied to valorize this waste sludge. Sludge composting and anaerobic sludge digestion are already widely applied at full-scale processes, but more options for sludge valorization are feasible and effective. Nevertheless, the integration of the water sector in the circular economy concept can only be performed if a close cooperation between governments, science, and commercial companies is targeted. More in-depth studies on cost analysis and safety could help increase the use of the proposed technologies as viable alternatives. Moreover, even though the market value of the recovered materials may not be high enough to justify its application, valorization strategies are more promising than traditional disposal methods, as they create value and reduce pollution.

## Figures and Tables

**Table 1:** Cities with wastewater resource recovery facilities. Adapted from [16].

City (Country)	Population 2016	Wastewater On-Site Sanitation/Sewer Service Coverage	Wastewater Treatment	Treated Wastewater Reused	Energy Recovered	Fertilizer Recovery	Potential to Reduce Emissions from Improved Wastewater Management
Aqaba (Jordan)	194,000	10/90%	100% 45 M. L/d	69%	100%	NO	81,000 ton CO_2_ eq/year
Bangkok (Thailand)	5.6 M	60/40%	100% 1.3 B L/d	5%	62%	YES	638,000 ton CO_2_ eq/year
Beijing (China)	21.7 M	5/95%	88% 4.4 B L/d	15%	45%	YES	1,044,000 ton CO_2_ eq/year
Chennai (India)	8.5 M	100%	70% 769 M L/d	49%	77%	NO	235,000 ton CO_2_ eq/year
Durban (South African)	3.7 M	84/16%	100% 108 M L/d	44%	8%	YES	438,000 ton CO_2_ eq/year
Kampala (Uganda)	1.5 M	60/40%	100% 87 M L/d	100%	227,000 kWh/y	YES	114,000 ton CO_2_ eq/year
Lima (Peru)	10 M	17/83%	15% 240 M L/d	5%	Low	NO	652,000 ton CO_2_ eq/year

**Table 2:** Industrial wastewater treatment processes aiming at water reuse.

Industrial Sector	Wastewater Source	Treatment	Reuse of the Treated Water	Reference
Aquaculture	Fish tanks	Mechanical filtration, biological filtration, oxygenation, and sterilization (ozone or UV)	Refeed the fish tanks	[19]
Brewery (beer)	Effluent from anaerobic digester	Flotation, membrane bioreactor, ultrafiltration, and reverse osmosis	Drinking water production	[20]
Agro-food (horticulture sector)	Mixture of water streams from vegetable processing, cleaning activities, and toilets	Oil removal, activated sludge process (with pre-denitrification scheme), sand filtration, membrane ultrafiltration, and UV sterilization	Irrigation of the own company fields	[21]
Dairy	Mixture of the powder (82%) and the butter (18%) effluent streams	Grease removal pond, anaerobic pond, and membrane bioreactor (ultrafiltration) with anoxic and aerobic zones.	Irrigation	[22]

**Table 3:** Summary of some actions from the National Water Reuse Action Plan in the United States.

Science and Specifications	Technology Development
Develop a list of constituents of concern and acceptable levels (or ranges) in potable water reuse.	Develop consistent approval processes and standards for new treatment technologies.
Develop guidelines for reviewing and permitting fit-for-purpose reuse applications.	Research science and technology gaps for onsite urban and stormwater reuse.
Research fit-for-purpose specifications and data gaps for oil and gas produced wastewater.	Research management and use for brine from reuse projects.
Develop a plan to manage and regulate high salt loads and disposal options from reuse water.	

**Table 4:** Microalgae culturing in wastewater streams in pilot-scale photobioreactors.

Stream Source	Type of Reactor	Biomass Productivity	Reference
Centrate resulting from sludge dewatering of urban WWTP in northern Italy	Raceway pond (Working volume of 1200 L)	5.5 ± 7.4 g TSS/m^2^/d	[42]
Centrate resulting from the solid fraction of piggery wastewater, energy crops, and agricultural waste co-digestion	Raceway pond (Working volume of 880 L)	8.2 g TSS/m^2^/d	[43]
Centrate resulting from sludge dewatering from a municipal Bresso WWTP, northern Italy	Column reactor (Working volume of 85 L)	50 mg TSS/L/d	[44]
Centrate resulting from sludge dewatering from a municipal Bresso WWTP, northern Italy	Bubble column reactor (Working volume of 82 L)	40 ± 62 mg TSS/L/d	[45]
Centrate resulting from sludge dewatering (20%) plus seawater	Tubular photobioreactors (Working volume of 340 L)	0.60 g biomass/L/d (at a dilution rate of 0.3/d)	[46]
Centrate resulting from sludge dewatering from a municipal WWTP plus crude glycerol (1 g/L)	Photobioreactor	460 mg TVS/L/d	[47]
Anaerobic digested starch processing wastewater	Airlift photobioreactor (Working volume of 890 L, 1.80 m length × 0.62/0.30 m breadth × 1.10 m height).	0.37 g/L/d	[48]
Raw dairy wastewater (25%)	Photobioreactors (Working volume of 40 L, 272 cm diameter × 1720 cm height)	110 mg/L/d	[49]
Dairy effluent	High-Volume V-shaped pond (working volume of 3 m^3^)	171 g/m^2^/d	[50]
Dairy farm wastewater	Single loop raceway (2.5 m × 0.7 m × 0.7 m and mixed by paddle wheel at 20 rpm)	0.38 ± 0.09 g/L/d	[51]
Swine wastewater (after grit removal and Canadian-type anaerobic digestion)	Raceway (Working volume of 15 L)	Up to 300 mg VSS/L	[52]
Swine manure (after pre-treatment to reduce the total suspended solid content by 70%) diluted 20- and 10-fold with tap water	Raceway (Working volume of 464 L with a surface of 1.54 m^2^ (2.3 m long × 0.70 m wide × 0.30 m deep)	Up to 21.3 and 27.7 g/m^2^/d (respectively)	[53]

TSS—total suspended solids; TVS—total volatile solids; VSS—volatile suspended solids.

**Table 5:** Single cell protein production from different streams at pilot-scale.

Waste Stream	Process	Culture	Productivity	Reference
Acidified stream from brewery industry	Aerobic with SRT < 8 days and nutrient addition	Enrichment of *Alpha-* and *Beta-proteobacteria*	>55% crude protein content	[56]
Mixture composed of CO_2_ and NH_3_ from sludge treatment plant and H_2_ and O_2_ from water electrolysis	Autotrophic by hydrogen using bacteria	H_2_-oxidizing bacteria	49–75% crude protein content 1 kg SCP/d	[57]
Domestic wastewater mixed with organic fraction of municipal solid waste	Anaerobic raceway	Purple phototrophic bacteria	n.r.	[58]

n.r.—not reported; SRT—sludge retention time.

**Table 6:** Nutrient and fertilizer recovery as valuable products from different kinds of wastewater at full-scale processes.

Product	Process	Stream	Recovery	Reference
Struvite	Crystallization	Reject water from sludge anaerobic digester (1.1–2.2 mmol PO_4_^−3^/L and 70 mmol NH_4_^+^/L)	77% of the Phosphorous Pellets of 0.5–5.0 mm	[65]
Ammonium sulphate	Vacuum stripping using gypsum and scrubbing	Reject water from sludge anaerobic digester (4.4 g NH_4_^+^-N/kg digestate)	57% of Ammonium	[66]
Ammonium sulphate	Pre-treatment with caustic soda, lamella clarification, filtration, and three-stage membrane contactor with sulphuric acid addition	Reject water from sludge anaerobic digester (1000 mg NH_4_^+^-N/L)	96% of Nitrogen removal efficiency and 4.1% of Nitrogen in the (NH_4_)_2_SO_4_ produced	[67]

**Table 7:** Energy recovery as biogas in anaerobic digestion processes using different feedstocks.

Feedstock	Scale	Type of Reactor	Biogas Productivity	Reference
Microalgae biomass plus primary sludge waste	Pilot-scale	Anaerobic membrane bioreactor	370 mL CH_4_/g VS _influent_	[77]
Waste activated sludge plus organic fraction of municipal solid waste	Full-scale	Pre-thickener plus a digester	up to 0.43 m^3^/kg TVS/d	[78]
Sewage sludge plus crude glycerol	Pilot-scale	Continuous stirred tank reactor	0.87 LCH_4_/g VS	[79]
Sewage sludge plus agro-industrial by-product (olive mill wastewater, crude glycerol, or cheese whey) (95/5, *v/v*).	Pilot-scale	Anaerobic digester	34.8 ± 3.2, 185.7 ± 15.3 and 45.9 ± 3.6 L/d, respectively	[80]
Screenings generated from the operations of pre-treatment of municipal wastewater	Pilot-scale	Mechanical stirring cylindrical digester (working volume of 50 L)	653 Nl/kg VS per week	[81]
Sewage sludge plus different beverage wastes (namely beer, soft drinks, fruit juice, or wine)	Pilot-scale	Anaerobic conical stainless steel reactor	Up to 237 L CH_4_/kg COD added	[82]
Municipal wastewater	Pilot-scale	Anaerobic membrane bioreactor	0.09–0.29 L CH_4_/g COD	[75]
Filtrate resulting of sludge dewatering	Pilot-scale	Upflow anaerobic sludge blanket reactor	260 mL/g COD	[83]

VS—volatile solids; TVS—total volatile solids.

**Table 8:** Configuration and performances of microbial fuel cells operation at pilot-scale.

Feedstock	Type of Reactor	Power Density Produced	Net Energy Recovered	Reference
Effluent of the primary clarifier of a WWTP	Four single-chamber membraneless MFCs (total volume of 45 L)	Up to 82 ± 18 mW/m^2^	Up to 0.025 ± 0.013 kWh/m^3^	[86]
Synthetic wastewater with variable influent COD concentrations (200–800 mg/L)	Five stacked MFC units(total volume of 72 L)	Up to 50.9 and 42.1 W/m^3^ in fed-batch and continuous, respectively	n.r.	[87]
Domestic wastewater from a WWTP	Single-chamber MFC unit	Up to 175.9 mW/m^2^	n.r.	[88]
Brewery wastewater	Five stacked MFC units(total volume of 90 L)	Up to 181 ± 21 mW/m^2^	0.097 kWh/m^3^	[89]
Municipal wastewater from a WWTP	50 stacked MFC units (total volume of 1000 L)	Up to 3.64 W/m^2^ (~60 W/m^3^)	0.033 ± 0.005 kWh/m^3^	[90]

n.r.—not reported.

**Table 9:** VFAs produced at pilot- and full-scale.

Waste Stream	Scale	Operating Conditions	VFA Production	VFA Composition (%)				Reference
				Acetic Acid	Propionic Acid	Butyric Acid	Others	
Primary sludge	Pilot scale	Sequencing batch fermentation reactor—2.6 m^3^ pH 6; HRT 6 days, 37 °C	154 ± 24 mg COD/g VS 1.23 Kg COD/m^3^_reactor_ d	30	45	-		[101]
		Sequencing batch fermentation reactor—2.6 m^3^ pH 6; HRT 14 days, 37 °C	137 ± 33 mg COD/g VS 0.44 Kg COD/m^3^_reactor_ d	25	53	-		
		Sequencing batch fermentation reactor—2.6 m^3^ pH 9; HRT 6 days, 37 °C	322 ± 56 mg COD/g VS 2.57 Kg COD/m^3^_reactor_ d	29	51	-		
Sewage sludge	Pilot scale	Stirred reactor coupled to a membrane separation system pH 5.7; HRT 5 days; SRT 14 days; 35 °C	206.5 mg COD/g TVS 3389 ± 1320 mg COD/L	31	28	23		[102]
		Stirred reactor coupled to a membrane separation system pH 10; HRT 6 days; SRT 14 days; 35 °C	315.6 mg COD/g TVS 7453 ± 1092 mg COD/L	40	24	17		
		Stirred reactor coupled to a membrane separation system pH 7; HRT 5 days; SRT 14 days; 35 °C	227.9 mg COD/g TVS 5596 ± 448 mg COD/L	42	30	15		
		Stirred reactor coupled to a membrane separation system pH 7; HRT 5 days; SRT 6 days; 35 °C	248.6 mg COD/g TVS 3738 ± 411 mg COD/L	42	30	16		
		Stirred reactor coupled to a membrane separation system pH 10; HRT 5 days; SRT 5 days; 35 °C	325.0 mg COD/g TVS 3184 ± 219 mg COD/L	50	16	10		
Sewage sludge	Full scale	Stirred tank reactor—30 m^3^ pH 10–11; 35 °C	261.32 mg COD/g VSS	58	7	-	35	[103]

HRT—hydraulic retention time; SRT—sludge retention time; VS—volatile solids; TVS—total volatile solids; VSS—volatile suspended solids.

**Table 10:** PHA production using mixed microbial cultures at pilot-scale.

Waste Stream	Operational Conditions	PHA Production	Polymer Produced	Reference
Milk and ice-cream wastewater	HRT = SRT 43.56 h; pH 7.1	0.25 Kg PHA/Kg COD _degraded_	PHA	[124]
Municipal wastewater and waste activated sludge	V reactor = 550 L; OLR = 3.0 g COD/L/d;HRT = 3 h;	0.25–0.38 g COD-PHA/g COD substrate0.27–0.38 g PHA/g VSS	Poly-(3HB-*co*-3HV)	[125]
Paper mill wastewater	V reactor = 200 L pH 6.6 T = 30 ± 2 °C	0.70–0.80 g PHA/g VSS	PHA	[126]
Municipal wastewater	V reactor = 511 L SRT = 0.28–0.56 days HRT = 30–60 min DO = 0.5–1.5 mg/L	26.3–51.4 mg COD-PHA/g VSS	PHB, PHV	[127]
Olive mill wastewater (OMW)	V reactor = 30 L T = 27 ± 2 °C	24.60 ± 0.21 g PHA/100 g VSS7.58 g PHA/L _initial OMW_	P3HB, P3HO or 3-HB-co-3-HO	[128]
Excess sludge fermentation liquid	V reactor = 70 L T = 30 °C DO > 80%pH not controlled	0.17 g PHA/g COD 6.497 mg PHA/L/h	PHA	[129]
Candy bar factory wastewater	V reactor = 200 L T = 30 ± 2 °C	0.76 g PHA/g VSS 0.30 ± 0.04 g COD-PHA/g COD	PHB, PHV	[130]
Municipal wastewater—anaerobic reject water	V reactor = 1 m^3^	0.40–0.44 g PHA/g VSS 0.58–0.61 g COD-PHA/g COD-VFA 224–234 mg PHA/L/h	PHB, PHV	[131]
Combined organic fraction of municipal solid waste and sewage sludge	V reactor = 50–70 L T = 22–25 °C pH = 8.0–9.0	0.43–0.46 g PHA/g VSS 0.44–0.50 g COD-PHA/g COD-VFA 0.29–0.36 g PHA/L/h	PHB, PHV	[132]
Activated sludge harvested from full scale municipal wastewater treatment—PHARIO	V reactor = 500 L T = 25 °C	0.41 g PHA/g VSS 0.40–0.45 g COD-PHA/g COD-substrate _consumed_	PHB, PHV	[133]

PHB—Polyhydroxybutyrate; PHV—Polyhydroxyvalerate; P3HB—Poly-3-hydroxybutyrate; P3HO—Poly-3-hydroxyoctanoate; 3-HB-co-3-HO—3-hydroxybutyrate-co-3-hydroxyoctanoate co-polymer.

**Table 11:** EPS recovery from granular sludge collected at pilot- and full-scale reactors.

Technology and Waste Stream	Scale	Operational Conditions	Extraction Method	Characteristics of Polymers	Yield	Reference
AGS—industrial diary wastewater	Full-scale SBR	Nereda^®^ process; n.r.	Heating method (Na_2_CO_3_, T = 80 °C)	Kaumera^®^ gum; n.r.	225 mg VSS _sEPS_/g VSS _AGS_	[154]
AGS—municipal wastewater	Full-scale SBR	Nereda^®^ process; COD tot,in = 585 mg/L; TSS,in = 195 mg/L; NH4-N,in = 55 mg/L; PO_4_-P,in = 6.3 mg/L; TSS,react = 8–10 g/L.	Heating method (Na_2_CO_3_, T = 80 °C)	69 ± 9% PolyGG blocks; 2 ± 1% PolyMM blocks; 15 ± 2% PolyMG blocks. Sodium alginate equivalent: 486 ± 22 mg Alginate/g VSS _sEPS_; Protein: <100 mg BSA/g VSS _sEPS_.	160 ± 4 mg VSS _sEPS_/g VSS _AGS_	[160]
AGS—municipal wastewater	Full-scale SBR	Nereda^®^ process; n.r.	Heating method (Na_2_CO_3_, T = 80 °C)	Polysaccharides: 138 mg Glucose/g VSS _sEPS_; Proteins: 381 mg BSA/g VSS _sEPS_;Uronic acid: 72 mg galact. acid/g VSS _sEPS_; Phenolic compound: 286 mg humic acid/g VSS _sEPS_.	282 mg VSS _sEPS_/g VSS _AGS_	[149,161,162]
AGS—mixed domestic, pluvial and industrial wastewater	Full-scale SBR	Nereda^®^ process; n.r.	Heating method (Na_2_CO_3_, T = 80 °C)	Polysaccharides: ≈ 10–15 mg Glucose/g VSS _sEPS_; Proteins: ≈ 60–80 mg BSA/g VSS _sEPS_; Humic substances: 317 mg humic acid/g VSS _sEPS_.	122–149 mg VSS _sEPS_/g VSS _AGS_	[155]
AGS—municipal wastewater	Pilot-scale SBR	COD tot,in = 461 mg/L; BOD5,in = 148 mg/L; TN,in = 43 mg/L; TP,in = 5 mg/L VER = 60%; SRT = 12–15 days.	Heating method (Na_2_CO_3_, T = 80 °C)	Polysaccharides: 136 mg Glucose/g VSS; Proteins: 514 mg BSA/g VSS.	219 mg VSS _sEPS_/g VSS _AGS_	[153]
AGS—wastewater from university campus	Pilot-scale SBR	VER = 60%; SRT= 10 d; COD tot,in = 1200 mg/L; TN,in = 52 mg/L; TP,in =12 mg/L.	Cation Exchange Resin (CER).	Polysaccharides: 224–252 mg Glucose/g VSS; Proteins: ≈11 mg BSA/g VSS.	n.r.	[163]
AGS—synthetic wastewater	Pilot-scale SBR	VER = 60%; COD tot,in = 8 g/L; TN,in = 450 mg/L; TP,in = 90 mg/L.	Thermal extraction (T = 80 °C)	Polysaccharides: 92 mg Glucose/g VSS; Proteins: 144 mg BSA/g VSS.	n.r.	[164]
Anammox	Full-scale	Two stage partial nitritation-anammox.	Alkaline extraction (NaOH)	Polysaccharides: 4–287 mg Glucose/g VSS; Proteins: 17–307 mg BSA/g VSS.	up to 380 mg VSS _sEPS_/g VSS _AMX_	[136,138,156,165]

n.r.—not reported; SBR—sequencing batch reactor; VER—volumetric exchange ratio; SRT—sludge retention time; GG—guluronic blocks; MM—mannuronic blocks; MG—heteropolymeric blocks.

**Table 12:** Applications of the recovered EPS in the different industrial sectors.

Application of EPS	Features	Reference
Coating material	The functional groups present in EPS provide abundant binding sites, both hydrophilic and hydrophobic functional groups, conferring improved waterproof capacity to surfaces.	[167,168]
Curing of cement	The hydrophilic properties of the EPS improve the curing of cement, reducing moisture loss from the surface of cement-based materials	[166]
Bioadsorbent	The physicochemical interactions between the adsorbates and functional groups of EPS, promote the adsorption of metals or other compounds	[170,171]
Flame retardant	sEPS can be extinguished bio-based flame retardant materials for flax fabrics due to effective char formation.	[173]
Bioflocculation	Some functional groups present on the EPS contribute to the flocculation abilities of these biopolymers	[174]
Soil conditioning	The water-binding capacity makes EPS applicable in the agronomic sector, as these biopolymers are able to retain water in soil and to reduce the leaching of fertilizers.	[175]
